# The Oncogene Transcription Factor EB Regulates Vascular Functions

**DOI:** 10.3389/fphys.2021.640061

**Published:** 2021-04-12

**Authors:** Gabriella Doronzo, Elena Astanina, Federico Bussolino

**Affiliations:** ^1^Department of Oncology, University of Torino, Candiolo, Italy; ^2^Laboratory of Vascular Oncology, Candiolo Cancer Institute-IRCCS-FPO, Candiolo, Italy

**Keywords:** angiogenesis, embryo, cell cycle, autophagy, inflammation

## Abstract

Transcription factor EB (TFEB) represents an emerging player in vascular biology. It belongs to the bHLH-leucine zipper transcription factor microphthalmia family, which includes microphthalmia-associated transcription factor, transcription factor E3 and transcription factor EC, and is known to be deregulated in cancer. The canonical transcriptional pathway orchestrated by TFEB adapts cells to stress in all kinds of tissues by supporting lysosomal and autophagosome biogenesis. However, emerging findings highlight that TFEB activates other genetic programs involved in cell proliferation, metabolism, inflammation and immunity. Here, we first summarize the general principles and mechanisms by which TFEB activates its transcriptional program. Then, we analyze the current knowledge of TFEB in the vascular system, placing particular emphasis on its regulatory role in angiogenesis and on the involvement of the vascular unit in inflammation and atherosclerosis.

## Introduction

The vascular unit is characterized by endothelial cells (ECs) lying on a basal membrane, where pericytes are embedded. Located at the interface between the bloodstream and tissues, the vascular unit orchestrates bidirectional information by integrating humoral, mechanical and cellular cues during the embryonic organogenesis process and in adults; this unit also plays a role in communicable and non-communicable diseases (Peng et al., 2019). Consequently, the vascular unit and, in particular, ECs undergo different genetic programs (Mantovani et al., 1997) regulated by specific transcription factors that adjust the transcriptional landscape to properly respond to different pathophysiological stimuli (De Val and Black, 2009; Park et al., 2013; Vanlandewijck et al., 2018).

Emerging evidence underlines autophagy as a key cellular mechanism involved in vessel development and physiological and pathological angiogenesis (Schaaf et al., 2019). Moreover, autophagy occurs on the basis of dynamic EC responses to changing environments, angiogenic cues, or intrinsic and extrinsic insults or injuries (such as metabolic stress, redox homeostasis and hypoxia). Activation of endothelial autophagy, as in other cell types, is mediated by different signaling pathways that are capable of regulating the autophagy-related gene complex, which controls various stages of the process as well as autophagosome formation and elongation, vesicle trafficking, and autophagosome lysosome fusion (Choi et al., 2013).

Transcription factor EB (TFEB) belongs to the microphthalmia (MiT) gene family of bHLH-leucine zipper transcription factors, which includes microphthalmia-associated transcription factor (MITF), TFE3 and TFEC. It was originally described to be translocated in a subset of renal carcinomas (Argani et al., 2005) and deregulated in melanomas and several carcinomas (Astanina et al., 2020). The current findings clearly support the existence of a canonical pathway by which TFEB acts as a master regulator of lysosomal and autophagosome biogenesis and represents a molecular tool to adapt cells to stress, including starvation and energy depletion (Sardiello et al., 2009; Napolitano and Ballabio, 2016; Saftig and Puertollano, 2020). TFEB directly promotes the transcription of some genes directly involved in autophagy (*ATG9, UVRAG, VPS11, VPS18, and WIPI)* and regulates autophagic flux (Settembre et al., 2011). Furthermore, it controls the expression of genes that orchestrate the expression, localization, entrance, influx, and performance of lysosomal and non-lysosomal enzymes participating in the destruction of cellular macromolecules (Sardiello et al., 2009; Palmieri et al., 2011; Settembre et al., 2011). In addition, TFEB can promote lysosomal exocytosis, allowing cargo secretion through fusion to the cell membrane (Medina et al., 2015).

In addition to the canonical pathway, new results definitively demonstrate wider regulatory activities encompassing metabolism, immunity, angiogenesis and inflammation, which are not necessarily associated with autophagy [see the following reviews: (Napolitano and Ballabio, 2016; Astanina et al., 2020; Irazoqui, 2020; Yu et al., 2020)] and refer to non-canonical pathways.

**FIGURE 1:**
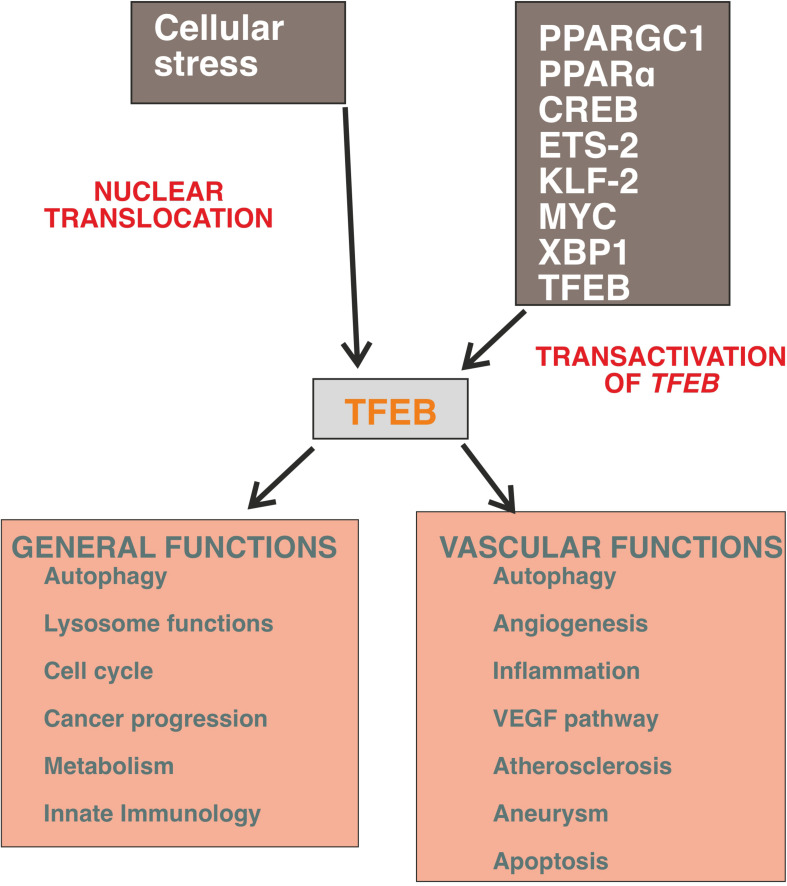
General and vascular activities of TFEB. The expression of TFEB is regulated by a several transcription factors and the most powerful stimulus to promote its nuclear translocation is mediated by cellular stress and cell starvation. The programs triggered by TFEB recognize the canonical autophagy pathway but it is emerging that this molecule regulates the cell transcriptional landscape independently from the control of autophagic flux (for details, see text).

This review summarizes our current understanding of the functions of TFEB in regulating EC activities in embryos and adults (Figure 1).

**TABLE 1:** Posttranslational modifications of TFEB *.

**Aminoacid Residue**	**Enzyme**	**Effect**
Ser 3	Phosphorylation by MAP3K3	Inhibit Ser 211 phosphorylation mediated by mTORC1
Ser 122	Phosphorylation by mTOR	Cytosolic retention
Ser 134	Phosphorylation by GSK3	Cytosolic retention
Ser 138	Phosphorylation by GSK3	Cytosolic retention and export from nucleus
Ser 142	Phosphorylation by mTOR, Erk 1/2	Cytosolic retention
Ser 142	Nuclear phosphorylation by CDK4 or CDK6	Nuclear export
Ser 211	Phosphorylation by mTOR	Cytosolic retention and 14-3-3 binding site
Ser 462	Phosphorylation by PKC	Stabilization of TFEB
Ser 463	Phosphorylation by PKC	Stabilization of TFEB
Ser 467	Phosphorylation by PKC	Stabilization of TFEB
Ser 467	Phosphorylation by AKT	Cytosolic retention
Ser 469	Phosphorylation by PKC	Stabilization of TFEB
Lys 116	Deacetylation by Sirtuin-1	Increase of transcriptional activity
Lys 116	Acetylation by GCN5	Suppression of transcriptional activity
Lys 274	Acetylation by GCN5	Suppression of transcriptional activity
Lys 279	Acetylation by GCN5	Suppression of transcriptional activity

*CDK4 and CDK6 also phosphorylate Ser 114 and Thr 331 but without any specific effect (Yin et al., 2020).

## Structure of TFEB

Transcription factor EB is a transcription factor with low tissue and cellular specificity that is widely expressed in fetuses and adults (Napolitano and Ballabio, 2016). The DNA-binding region is characterized by a helix-loop-helix (HLH) and a leucine zipper domain (Zip) flanked by an upstream basic region that is able to recognize an E-box sequence (CAYGTG) in the promoter regions of targeted genes (Carr and Sharp, 1990; Fisher et al., 1991; Palmieri et al., 2011; Doronzo et al., 2019). The Zip domain is essential for homodimerization or heterooligomerization with other MiT genes. The TFEB structure is also characterized by a glutamine- and proline-rich domain, which has poorly described functions that are discussed in Box 1.

**FIGURE 2:**
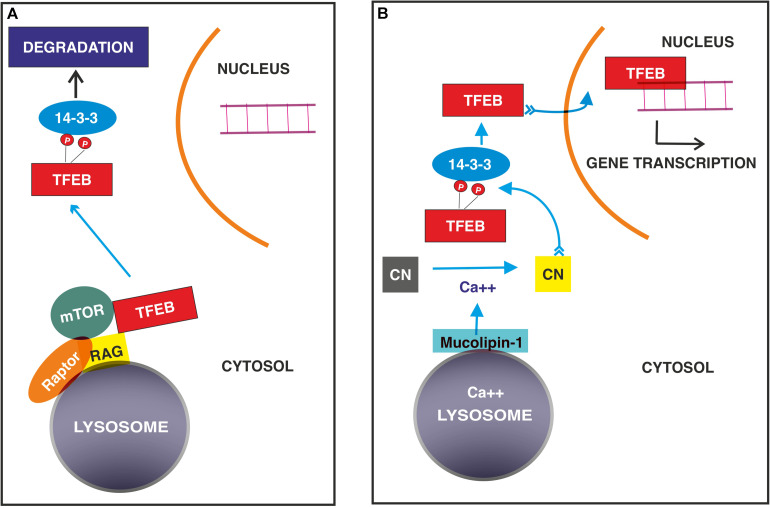
Signaling mechanisms regulating TFEB nuclear translocation in normal **(A)** and stressed conditions **(B)**. **(A)** TFEB binds GTP- RagGTPase on the surface of lysosome followed by the recruitment of Raptor and mTOR. This complex allows the phosphorylation of TFEB, which interacts with 14-3-3 adaptor with subsequent degradation. **(B)** In stressed conditions, calcium ions released from lysosomes through mucolipin 1 channel activates the protein phosphatase calcineurin, (CN) which dephosphorylates TFEB, allowing its nuclear translocation.

**Box 1. Structure of TFEB (Figure 2).**

TFEB has been highly conserved throughout evolution and is present in flies, fishes, avians, and mammals (Steingrímsson et al., 1998; Hallsson et al., 2004; Lister et al., 2011; Lapierre et al., 2013), exhibiting similar genomic organization and structural features. Human TFEB is constituted by 476 amino acids and has a mass of ∼53 kDa. *TFEB* spans approximately 51,000 bp and is located on chromosome 6, whereas mouse Tfeb (475 amino acids) is on chromosome 17 and extends over 55,000 bp. Genomic organization analysis of the human gene unveiled the presence of nine exons, each of which is spliced to the common coding exons 2–9 (Kuiper et al., 2004). This organization generates a 2,364 bp mRNA transcript consisting of two non-coding exons and eight coding exons, with a 302-bp 5′ UTR followed by a start codon in exon 3 and a stop codon in exon 10, followed by a 621 bp 3′ UTR. Seven alternative mRNAs with the same translational start site at exon 2 have been described with differential and restricted tissue distributions (Kuiper et al., 2004; Vu et al., 2021). The mouse TFEB protein is 94% identical to its human ortholog, sharing domain structure organization and posttranslational modification sites (Steingrímsson et al., 2004).

As the other member of the MiT gene family, the bHLH (position 235–288) and Zip (position 298–319) domains establish the DNA binding region. The activation domain (AD) is mapped at position 156–165 and predicts a binding site for the transcriptional coactivator p300 (Muhle-Goll et al., 1994). In the absence of DNA, a tetramer-sized form of TFEB is formed that dissociates to bind added DNA as a dimer (Fisher et al., 1991; Muhle-Goll et al., 1994). The TFEB structure also contains a N-terminus glutamine-rich region (position 10–44) encompassing the binding site of Rag C and a C-terminal proline-rich region (position 366–414) with undetermined functions. Of note, this domain is also present in TFE3, where it has activating functions (Artandi et al., 1995). The abilities of TFEB to promote autophagy and lysosome biogenesis are mediated by a palindromic consensus sequence (GTCACGTGAC) overlapping the E-Box sequence, named the coordinated lysosomal expression and regulation motif (CLEAR) (Sardiello et al., 2009; Palmieri et al., 2011; Settembre et al., 2011).

## Mechanisms Involved in TFEB Activation

Transcription factor EB is localized in the cytosol in an inactive state induced mainly by the phosphorylation of specific amino acid residues and translocates to the nucleus to start specific transcriptional programs when dephosphorylated (Figure 2).

The expression of TFEB is transcriptionally regulated by several factors, including androgen receptor (Shang et al., 2020), peroxisome proliferator-activated receptor-γ coactivator 1α (PPARGC1α), retinoid X receptor-α (RXRα), the peroxisome proliferator-activated receptor-α (PPARα) complex (Salma et al., 2017; Byun et al., 2020), cAMP response element-binding protein (CREB), the CREB-regulated transcription coactivator 2 complex (Seok et al., 2014), ETS2 (Ma et al., 2016), Krüppel-like factor 2 (KLF2) (Song et al., 2019), MYC (Annunziata et al., 2019), XBP1 (Zhang Z. et al., 2020) and TFEB itself (Settembre et al., 2011).

Interestingly, some of these transcription factors (PPARGC1α, CREB, and MYC) are involved in the metabolic regulation and reprogramming observed in stressed conditions and cancer (Stine et al., 2015; Dubois et al., 2017; Berdeaux and Hutchins, 2019).

A wide number of transcription factors promoting TFEB activation indicate that it might participate in many biological functions, but the external cues regulating TFEB transcription and activation are poorly investigated. However, the available findings strongly indicate that TFEB regulates energy homeostasis under the direct or indirect control of the two metabolic sensors mTOR and AMP-activated protein kinase (AMPK) both in physiological and pathologic settings.

The most defined system, which restricts TFEB to the cytosol, thus blocking its nuclear translocation, is represented by mTORC1 (Roczniak-Ferguson et al., 2012; Settembre et al., 2012; Palmieri et al., 2017; Vega-Rubin-de-Celis et al., 2017) and Rag GTPases, which localize both mTORC1 and TFEB itself on the cytosolic surface of lysosomes and degrade TFEB (Settembre et al., 2012; Martina and Puertollano, 2013; Petit et al., 2013; Di Malta et al., 2017). Therefore, when cells are fed, activated mTORC1 regulates anabolic pathways and phosphorylates TFEB, impeding its nuclear translocation.

Activated protein kinase exerts a mirrored function. In the absence of nutrient availability, AMPK promotes the dephosphorylation of TFEB and its translocation to the nucleus (Collodet et al., 2019; El-Houjeiri et al., 2019). At present, this dephosphorylation mechanism is unknown.

In addition to phosphorylation, other posttranslational modifications, including dephosphorylation (Settembre et al., 2011, 2012; Roczniak-Ferguson et al., 2012; Ferron et al., 2013; Medina et al., 2015; Li et al., 2016; Palmieri et al., 2017; Vega-Rubin-de-Celis et al., 2017; Hsu et al., 2018; Martina and Puertollano, 2018; Zhang et al., 2018), acetylation/deacetylation (Miller et al., 2005; Bao et al., 2016; Wang et al., 2020) and sumoylation (Napolitano et al., 2020), regulate nuclear-cytosolic shuttling and nuclear activity (Box 2).

**Box 2. Posttranslational modifications regulate TFEB transcriptional activity (Table 1).**

**TFEB phosphorylation**

TFEB phosphorylation is required for the control of both nuclear entry and export. The phosphorylation of Ser 122, 142, and 211 (Settembre et al., 2011, 2012; Martina et al., 2012; Roczniak-Ferguson et al., 2012; Vega-Rubin-de-Celis et al., 2017) is required for TFEB degradation, while Ser 462, 463, 466, 467 and 469 likely favor its activation, as inferred from the observation that the substitution of these residues with phosphomimetic aspartate forces TFEB nuclear translocation (Peña-Llopis et al., 2011). Many phosphorylation events occur at the lysosomal surface and are mediated by mTORC1. (Roczniak-Ferguson et al., 2012; Settembre et al., 2012; Palmieri et al., 2017; Vega-Rubin-de-Celis et al., 2017). The first 30 amino acid residues of TFEB bind Rag GTPase in the GTP active binding configuration (Martina and Puertollano, 2013) and allow its localization on the cytosolic membrane of lysosomes (Settembre et al., 2012; Martina and Puertollano, 2013; Petit et al., 2013; Di Malta et al., 2017). This interaction is crucial for the recruitment of mTOR and Raptor (regulatory associated protein of mTOR) and promote mTOR-dependent TFEB phosphorylation.

Once Ser 211 is phosphorylated, TFEB is released from the lysosomal surface and bound by the 14-3-3 scaffold protein, rendering it inactive in the cytosol (Settembre et al., 2011, 2012; Roczniak-Ferguson et al., 2012; Vega-Rubin-de-Celis et al., 2017). Phosphorylation of Ser 142 and 211 induce the degradation of TFEB through the ubiquitin-proteasome pathway (Sha et al., 2017), while Ser 122 enhances the effect of phosphorylated Ser 211 (Vega-Rubin-de-Celis et al., 2017).

In low nutrient conditions, the Rag GTPase in the GTP-active configuration is inactivated by two GTPase-activating proteins named GATOR 1 and Folliculin, resulting in the release of mTOR in the cytosol (Saxton and Sabatini, 2017).

In addition to mTOR, extracellular signal-regulated kinase (ERK) 2, mitogen-activated protein kinase kinase kinase 3 (MAP3K3), glycogen synthase kinase (GSK)3β, protein kinase Cβ (Ferron et al., 2013) and AKT (Palmieri et al., 2017) recognize TFEB as a substrate and differentially regulate its fate. ERK2 (Settembre et al., 2011, 2012), GSK 3β (Li et al., 2016) and AKT (Palmieri et al., 2017) contribute to TFEB retention in the cytosol, protein kinase Cβ stabilizes TFEB and cooperates with mTOR (Ferron et al., 2013), and MAP3K3 antagonizes TFEB phosphorylation by mTORC1(Hsu et al., 2018).

A particular mechanism is played by phosphatase calcineurin, which is activated by calcium released from lysosomes in stressed conditions and dephosphorylates TFEB at Ser 211 and 142, thus inducing its nuclear translocation (Medina et al., 2015). Similarly, protein phosphatase 2A activated by oxidative stress dephosphorylates TFEB at the Ser 109, 114, 122 and 211 residues (Martina and Puertollano, 2018.

Phosphorylation is also involved in regulating the export of TFEB from the nucleus to the cytosol when its transcriptional activity needs to be interrupted. A hydrophobic nuclear export sequence has been mapped at residues 129–152 and encompasses Ser 142 and 138. Interestingly, the phosphorylation of Ser 142 by mTOR or ERK2 primes the nuclear export sequence for phosphorylation by GSK β at Ser 138, achieving an efficient nuclear export through exportin-1 (Li et al., 2018; Napolitano et al., 2018). Finally, TFEB activates the transcription of cyclin−dependent kinase (CDK) 4 (Doronzo et al., 2019), which may phosphorylate TFEB at Ser 142, thus allowing its nuclear export (Yin et al., 2020) and damping TFEB-mediated cell cycle activation (Brady et al., 2018; Doronzo et al., 2019).

**TFEB acetylation and deacetylation**

TFEB contains some Lys residues, which are substrates of acetylating and deacetylating enzymes, and acetylation appears to inhibit TFEB dimerization and its capability to bind the promoter regions of target genes (Wang et al., 2020). In microglia, the deacetylating enzyme sirtuin-1 binds and deacetylates TFEB at the Lys116 residue, thus increasing its transcriptional function (Bao et al., 2016). In contrast, in cancer cell lines, inhibitors of histone deacetylases indirectly favor acetylation of Lys 91, 103, 116, and 430 at the nuclear level (Zhang et al., 2018) and increase TFEB transcriptional activity. Finally, it has been reported that histone deacetylase can bind the *TFEB* promoter to inhibit its expression (Annunziata et al., 2019). The data available are partially conflicting and might suggest that the effects of these posttranslational modifications depend on the cellular context.

## A Paradigmatic Example of the Multifaceted Role of TFEB in Pathology: Cancer

The regulatory role of mTOR and AMPK on TFEB is part of the complex metabolic scenario occurring in cancer. TFEB is deactivated by mTORC1 under the condition of nutrient availability *via* its cytoplasmic retention but also controls mTORC1 lysosomal recruitment, which is required for its activation, creating a mechanism for transducing the information of the cell energy environment into the switch between anabolic and catabolic pathways (Perera et al., 2015; Di Malta et al., 2017; Li et al., 2019). Upregulation of TFEB in several cancers, such as pancreatic adenocarcinoma, melanoma, renal cell carcinoma colorectal cancer and non-small cell lung cancer (Davis et al., 2010; Liang et al., 2010; Giatromanolaki et al., 2015; Li et al., 2019), promotes cancer progression *via* mTORC1 hyperactivation signaling to promote cell proliferation and boost autophagy, producing an intracellular pool of nutrients, most importantly amino acids, and thus supporting cancer growth. Moreover, in pancreatic cancer, TFEB-induced autophagic activation leads to cancer progression *via* increased migration and metastasis of cancer cells due to endocytosis of Itgα5 and disassembly of adhesion machinery (He et al., 2018). The autophagy-independent function of TFEB was demonstrated in breast cancer, where TFEB inhibited apoptosis by regulating DNA repair mechanisms (Slade et al., 2020). More evidence supports a role for TFEB in the tumor microenvironment. In tumor-associated macrophages, TFEB acts as a major switch of both canonical and non-canonical pathways, leading to attenuation of the tumor-supporting phenotype. TFEB inhibits STAT3 activation, thus suppressing the production of an array of tumor-associated macrophage effector molecules and blocking the transcription of PPARγ, inhibiting the downstream expression of proinflammatory cytokines and HIF1α. Moreover, TFEB-induced increases in autophagic and lysosomal activity disactivate inflammasomes and degrade the HIF1α protein, thereby halting the hypoxic response associated with cancer progression (Li et al., 2020).

## TFEB Regulates Vascular Functions

### TFEB and Angiogenesis in Embryos and Adults

Mouse mutants have suggested that Tfeb functions as a modulator of endothelial activities related to angiogenesis in both embryos (Steingrímsson et al., 1998; Doronzo et al., 2019) and adult animals (Fan et al., 2018; Doronzo et al., 2019).

In wild-type mice, Tfeb is expressed at low levels in E8.5- to 10.5-day-old embryos, while it is highly expressed in labyrinthine trophoblasts from 8.5-day-old placentas. In *Tfeb* null mice, vascular invasion of the labyrinthine trophoblast layer by the embryo is blocked, and capillaries stop in the chorion. In contrast, maternal sinuses invade the placenta, although they are fewer and smaller than normal. Other defects of extraembryonic tissue are not evident. Interestingly, these placental vascular defects precede embryonic lethality, suggesting that the observed lethality is caused by placental defects, which are then instrumental in inducing hypoxia and cell necrosis (Steingrímsson et al., 1998). Mechanistically, *Tfeb* null mice express lower trophoblast levels of vascular endothelial growth factor (VEGF) A than wild-type mice, supporting that Tfeb directly or indirectly controls the expression of this angiogenic inducer (Steingrímsson et al., 1998).

The specific endothelial deletion of *Tfeb* is lethal at E10.5, with vascular alterations explained by defects in the remodeling of the primitive vascular plexus. Vessels appear irregular and dilated with reduced branching and impairment of invasion into intersomitic tissue. Furthermore, the yolk sac appears poorly vascularized and exhibits a hypoxic area (Doronzo et al., 2019). When *Tfeb* is deleted in all tissues (Steingrímsson et al., 1998), homozygous null mice die between E9.5 and E11.5 and exhibit characteristics of general and diffuse cell damage, particularly at somites, neural tubes, ganglia and mesenchyme.

**FIGURE 3:**
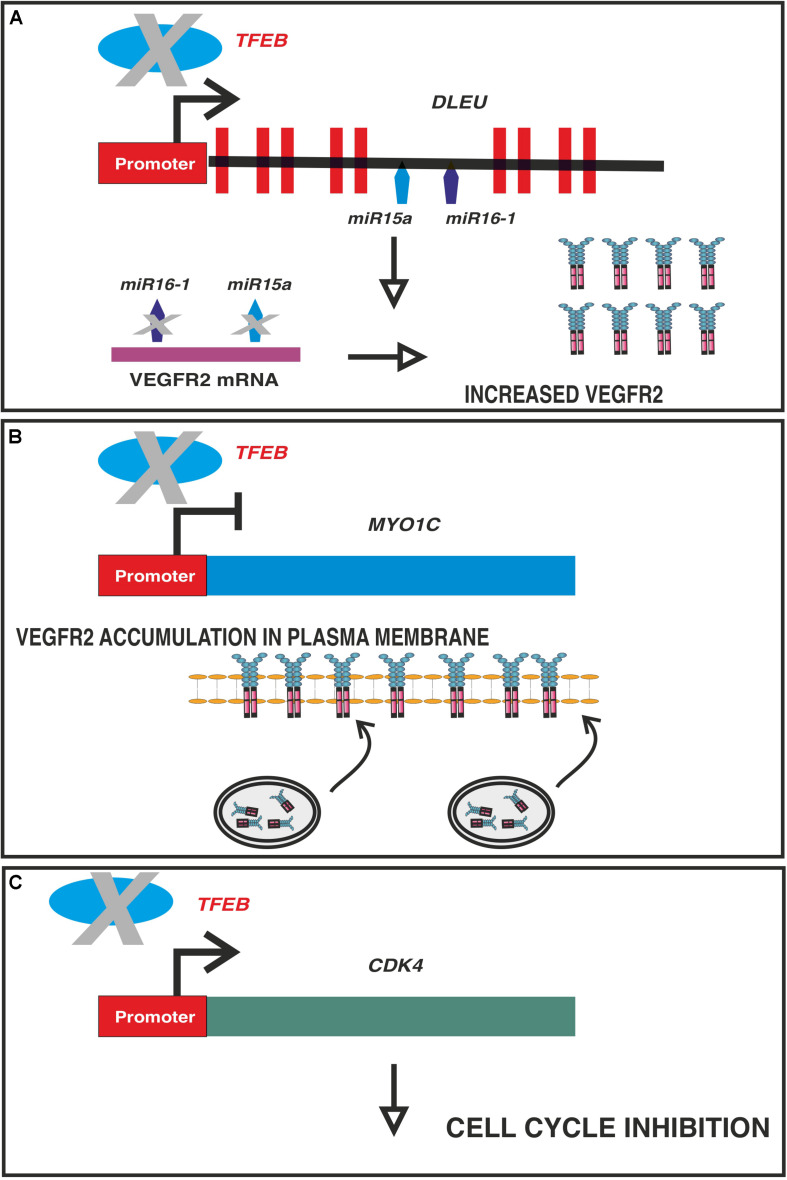
Multiple effects of TFEB deletion on ECs. In vascular ECs, TFEB directly binds and regulate the promoters of: **(A)** *DLEU2*, which encodes miR-15a/16-1 cluster **(A,B)** *MYO1C* and **(C)** *CDK4*. Panel **(A)**: in absence of TFEB the post-transcriptional regulation of VEGFR2 mediated by miR-15a-5p and miR-16-5p is impaired leading to an increased stabilization of the transcript. Panel **(B)**: TFEB is a repressor of *MYO1C*, an unconventional myosin involved in VEGFR2 exocytosis. In absence of TFEB, the expression of this gene is increased and more VEGFR2 is available and transported to plasma membrane through Rab4 ^+^ exocytic vesicles. As a consequence the receptor recycling is impaired with a reduced efficiency of signaling machinery. Panel **(C)**: TFEB transactivates *CDK4* and the consequence of its deletion is the cell cycle in G1 phase.

Alterations in the VEGF pathway were further highlighted in conditional and endothelium-specific Tfeb knockout mice. In this model, endothelial *Tfeb* deletion after birth results in impairment of the maturation of retinal and renal vasculature because the EC cycle is blocked by the reduced expression of cyclin−dependent kinase (CDK) 4, which is under the direct transcriptional control of TFEB (Doronzo et al., 2019). As a consequence, the phosphorylation of the retinoblastoma protein is reduced, blocking the nuclear translocation of E2F to transcribe genes necessary for the S−phase of the cell cycle (Doronzo et al., 2019). Of note, the ability of TFEB to regulate the cell cycle independent of the autophagy pathway is not restricted to ECs, as it is observed in other cell types (Brady et al., 2018; Pastore et al., 2020; Pisonero-Vaquero et al., 2020). In an attempt to restore the cell cycle, ECs lacking TFEB overexpress VEGF receptor (R)2, but this process is ineffective in restoring cell proliferation. This cell response is not mediated by a direct effect of TFEB on VEGFR2 transcription because its promoter does not exhibit any specific binding site. In contrast, TFEB transactivates the intragenic miR-15a/16-1 cluster, which limits the stability of the VEGFR2 transcript (Chamorro-Jorganes et al., 2011; Chan et al., 2013). In the absence of this posttranscriptional regulatory mechanism, *VEGFR2* mRNA is stabilized, thus promoting receptor accumulation (Doronzo et al., 2019). However, the signaling properties of VEGFR2 rely on not only the catalytic activity of the kinase domain but also its trafficking, which is instrumental for its performance (Simons et al., 2016). TFEB represses *MYO1C*, an unconventional myosin protein, which promotes VEGFR2 delivery to the plasma membrane (Tiwari et al., 2013). The regulated activity of VEGFR2 in ECs requires its internalization, trafficking to endosomes, and either transport to lysosomes for degradation or recycling back to the plasma membrane (Simons et al., 2016). In TFEB knockdown cells, the reduced and increased amounts of miR-15a/16-1 and MYO1C, respectively, result in the overexpression of VEGFR2 on the plasma membrane, which shows low signaling activity (Doronzo et al., 2019) that is most likely dependent on defects in receptor trafficking. Figure 3 summarizes the multiple effects induced by TFEB deletion in ECs.

In ECs, autophagic flux is controlled at the transcriptional level by NF-kB (Zeng et al., 2016; Leonard et al., 2019) and FOXO (Liu et al., 2015). It has also been recently reported that TFEB controls autophagy in ECs. This activity has been associated with the angiogenic response in ischemic skeletal muscle. Capillary ECs overexpressing TFEB respond to limb ischemia by promoting angiogenesis and improving tissue blood perfusion. In contrast, this proangiogenic activity is blunted in ECs devoid of TFEB. The angiogenic response is mediated by an increase in autophagic genes and activation of AMP-activated protein kinase signaling, as inferred by pharmacological inhibition or genetic silencing (Fan et al., 2018). The role of autophagy in angiogenesis represents a mechanism by which ECs can accommodate the metabolic changes required to support their migration and proliferation (Schaaf et al., 2019). Furthermore, autophagy represents a mechanism by which angiostatic signals are transferred from the extracellular matrix. For instance, the secreted proteoglycan decorin exerts antiangiogenic activity by activating autophagy in ECs (Torres et al., 2017), and this effect is mediated by TFEB. In this context, the nuclear translocation of TFEB relies on the catalytic activity of VEGFR2, as inferred by the use of tyrosine kinase inhibitors, which halt decorin-mediated TFEB activation (Neill et al., 2017).

### TFEB Controls an Inflammatory Program in ECs

Recently, some studies have highlighted the anti-inflammatory role of TFEB in ECs (Lu et al., 2017; Song et al., 2019). Laminar shear stress, known to protect against atherosclerosis but not oscillatory shear stress, induces TFEB activation. ECs exposed to laminar flow express more TFEB than those exposed to static conditions by a mechanism mediated by Krüppel-like factor 2, which is a shear stress-responsive factor. TFEB overexpression results in a reduction in the synthesis of inflammatory cytokines and adhesive molecules and in the adhesiveness of circulating monocytes in response to an inflammatory stimulus. This phenotype is reverted by TFEB knockdown. These effects are independent of autophagic flux but rely on the suppressive activity of the NF-kB pathway. TFEB inhibits IκB kinase activity, leading to reduced p65 nuclear translocation (Song et al., 2019). Furthermore, the overexpression of TFEB in human ECs isolated from cord veins transactivates the antioxidant enzymes heme oxygenase-1 and superoxide dismutase 2 (Lu et al., 2017). In parallel, the overexpression of TFEB reduces the production of radical oxygen species, whereas TFEB knockdown has the opposite effect (Lu et al., 2017). A similar event was reported in cardiac endothelial cells subjected to ischemia/reperfusion both *in vitro* and *in vivo* (Zhang Y.J. et al., 2020). However, in this model, NAD^+^-dependent TFEB expression recovers the autophagic flux damaged by ischemic injury and prevents apoptosis. The antiapoptotic effect of TFEB was further investigated in vascular smooth muscle cell (VSMC)-selective *Tfeb* knockout mice and in human and mouse aortic aneurysm samples. TFEB binds the promoter of BCL2 in VSMCs, enhances its transcription and halts apoptosis. However, in this experimental setting, the antiapoptotic effect of TFEB was independent of autophagic flux because knockdown of the autophagic gene *ATG7* did not abolish its antiapoptotic effect (Lu et al., 2020).

### TFEB and the Atherosclerotic Process

Autophagy in both vascular ECs and VSMCs is a protective and prosurvival mechanism aimed at combating many pathophysiological stimuli, including oxidized low-density lipoproteins, reactive oxygen species, inflammatory stimuli, shear stress and hypoxia (De Meyer et al., 2015; Jiang, 2016). However, autophagy can exert a dangerous effect when the pathological stimulus burden is dominant (De Meyer et al., 2015; Jiang, 2016).

A paradigmatic example of this bimodal function in vascular pathology is atherosclerosis (Martinet and De Meyer, 2009; De Meyer et al., 2015). Autophagy is involved in promoting reverse cholesterol transport from macrophages infiltrating the vessel wall and protecting plaque cells from middle injuries, thereby favoring plaque stability (Ouimet et al., 2011). However, when oxidative stress and turbulent blood flow worsen, the autophagic response is insufficient to remove altered macromolecules and organelles (e.g., mitochondria). Damage to mitochondria and lysosome membranes results in the release of cytochrome c, mtDNA (Tumurkhuu et al., 2016) and acidic hydrolases (Wen and Weak, 2007) and in the subsequent activation of apoptosis, impairment of autophagosome formation and accumulation of insoluble ceroids constituted by proteins precipitated with oxidized lipids (Wen and Weak, 2007). The compromised autophagic flux in ECs and infiltrating macrophages results in the reduction of antithrombotic function of the vessel wall and in plaque instability.

In the atherosclerotic process, TFEB activation likely aims to revert the impairment of autophagic flux, as described in lysosomal storage diseases (Parenti et al., 2015). The potential role of TFEB in eliminating insoluble ceroids was demonstrated both *in vitro* and *in vivo*. Specifically, induction of lysosomal biogenesis by overexpression of TFEB in macrophages rescues ceroid-induced lysosome dysfunction, defers inflammasome activation, enhances cholesterol efflux, and reduces the *in vivo* progression of atherosclerotic disease in proatherogenic ApoE-null mice (Emanuel et al., 2014; Sergin et al., 2017). Interestingly, sorting nexin 10, a protein involved in regulating endosome trafficking, accumulates in atherosclerotic plaques and inhibits TFEB nuclear translocation by promoting AKT-dependent phosphorylation. Deletion of this protein in atherosclerotic macrophages suppresses the AKT pathway and increases the nuclear translocation of TFEB (You et al., 2020).

The inhibitory effect of TFEB on the atherosclerotic process was confirmed by findings obtained in VSMCs. In this cell type, oxidized lipoproteins inhibit the transcription of TFEB, which is counteracted by overexpression of stearoyl−coenzyme A desaturase−1, an integral protein anchored in the endoplasmic reticulum membrane. Overexpression of this enzyme inhibits the differentiation of VSMCs in foam cells and increases TFEB nuclear translocation (Pi et al., 2019). In mice maintained on a high-fat diet and subjected to partial carotid ligation, VSMCs undergo neointima formation, which is a hallmark of atherosclerotic plaques. The proliferation of VSMCs is dependent on the inhibition of TFEB-mediated autophagic flux and blocked by TFEB reactivation (Wang et al., 2019).

## Conclusion and Perspectives

Current knowledge regarding the TFEB regulation of the vascular system is in its infancy, but the emerging results regarding its molecular and biological activities in other cellular types envisage its potential effects on many physiopathological conditions involving the vasculature, from cancer to inflammatory, metabolic and neurodegenerative diseases. However, to truly understand the budding role of TFEB in vascular medicine, several issues must be faced and solved. First, it is necessary to increase our knowledge of TFEB in vascular processes characterized by the activation of autophagy, such as pulmonary arterial hypertension, Fabry disease, pathological angiogenesis, atherosclerosis, chronic and acute ischemic disorders, and aneurysms. Because autophagy is associated with the endoplasmic reticulum stress response, which has an emerging role in vascular homeostasis (Battson et al., 2017), it might be relevant to understand how this stress response is regulated by TFEB in the vasculature. Interestingly, TFEB has been reported to transactivate the unfolded protein response and activate transcription factor 4 in retinal cells (Martina et al., 2016), which are key players in this pathway.

Second, TFEB is likely a hub of an undefined transcription factor network, which dynamically shapes the transcriptional landscape in a cellular context-dependent manner. To date, these transcriptional circuits have been poorly investigated (Astanina et al., 2020) and raise the question of whether other genetic programs are orchestrated or modulated by TFEB in addition to the well-established canonical autophagy process. Furthermore, elucidating the correlation between the level of TFEB activation in the vasculature and the type of transcriptional response is important to understand the extent to which TFEB will be a druggable target. A paradigmatic example is provided by hypoxia-inducible factor, which determines the features of gene expression according to the harshness of hypoxia (Pouysségur et al., 2006). Third, new findings on the effects of TFEB on key mechanisms of vascular homeostasis, such as endothelial metabolism, endothelial-mesenchymal transition, the cell cycle and cell motility, might open new translational opportunities.

## Author Contributions

EA wrote the sections on the general aspect of TFEB biology. FB inspired the review and supervised the final version. GD wrote the sections related to the vascular functions of TFEB. All authors contributed to the article and approved the submitted version.

## Conflict of Interest

The authors declare that the research was conducted in the absence of any commercial or financial relationships that could be construed as a potential conflict of interest.
